# The Canadian Breast Cancer Symposium 2025: Meeting Report

**DOI:** 10.3390/curroncol33010015

**Published:** 2025-12-27

**Authors:** Christine Brezden-Masley, Katarzyna J. Jerzak, Nancy A. Nixon, Anne Koch, Amanda Roberts, Jean-François Boileau, May Lynn Quan, MJ DeCoteau, Tulin D. Cil

**Affiliations:** 1 Marvelle Koffler Breast Centre, Mount Sinai Hospital, University of Toronto, Toronto, ON M5G 1X5, Canada; 2 Sunnybrook Odette Cancer Centre, University of Toronto, Toronto, ON M4N 3M5, Canada; 3 Department of Surgery and Oncology, University of Calgary, Calgary, AB T2N 4Z6, Canada; nancy.a.nixon@ahs.ca (N.A.N.);; 4 Princess Margaret Cancer Centre, Toronto, ON M5G 2M9, Canada; 5 Jewish General Hospital, Montreal, QC H3T 1E2, Canada; 6 Rethink Breast Cancer, Toronto, ON M4M 3G3, Canada

**Keywords:** breast cancer, patient voice, systemic therapy, new advances, CNS metastases, surgical oncology

## Abstract

The 2025 Canadian Breast Cancer Symposium (CBCS) brought together patients, clinicians and researchers from across Canada to discuss advances shaping personalized breast cancer care. Key updates in systemic therapy highlighted expanding treatment options, including CDK4/6 inhibitors, oral SERDs, PI3K/AKT-targeted therapies, and antibody–drug conjugates across early and metastatic settings. Radiation oncology sessions emphasized treatment de-escalation, featuring evidence for ultra-hypofractionation, selective omission of nodal irradiation, and stereotactic strategies to manage oligoprogression. Surgical presentations focused on reducing morbidity through tailored axillary management and emerging techniques to prevent lymphedema. Advances in the management of central nervous system metastases underscored the growing synergy between stereotactic radiotherapy and CNS-active systemic therapies. Informed by patient testimony and advocacy perspectives, experts reflected on persistent gaps in diagnosis, access, and survivorship that shape priorities for future improvements. Together, these insights outline key directions that help to refine clinical practice and guide future research.

## Simple Summary

This report summarizes key insights from the 2025 Canadian Breast Cancer Symposium, where patients, clinicians, and researchers came together to share the latest advances in breast cancer care. Participants highlighted the growing importance of personalizing treatment—from improving early detection and imaging to refining surgery, radiotherapy, and systemic therapies for different breast cancer subtypes. Patient stories emphasized ongoing gaps in diagnosis and the need for more equitable, timely, and compassionate care. Experts also reviewed emerging tools such as circulating tumour DNA, new targeted therapies, and innovative imaging technologies that may transform how breast cancer is monitored and treated in the future. Discussions about global health, survivorship, and advocacy underscored that scientific progress must be paired with patient-centred approaches. Overall, the symposium outlined current challenges, showcased practice-changing research, and identified priorities that could shape future studies, inform policy, and improve real-world outcomes for people with breast cancer.

## 1. Introduction

The Canadian Breast Cancer Symposium (CBCS) remains a leading national, multidisciplinary event committed to advancing personalized breast cancer care. Held on the 19–20th June 2025 at the Hilton Hotel in Toronto, Ontario, the symposium brought together a diverse community of oncologists, surgeons, pathologists, nurses, residents and fellows, patient advocates, and survivors from across Canada. Chaired by Christine Brezden-Masley, MD, PhD, FRCPC (Mount Sinai Hospital, University of Toronto, Toronto, ON, Canada) and Tulin Cil, MD, MEd, FRCSC, FACS (Princess Margaret Cancer Centre, University Health Network, Toronto, ON, Canada), this year’s conference featured 15 plenary sessions designed to tackle both longstanding challenges and emerging frontiers in breast cancer care (Table S1).

Throughout the symposium, experts highlighted the growing importance of patient-centred approaches, including discussions on pregnancy and breast cancer, survivorship, and advocacy efforts that are reshaping care delivery. Key sessions also explored global perspectives and presented the top breast cancer research papers of 2025, offering insight into the evolving evidence guiding clinical decisions worldwide. With a focus on innovation, speakers also examined cutting-edge diagnostic tools and technologies, such as the potential integration of circulating tumour DNA (ctDNA) in early-stage disease and advances in radiology, breast screening, radiation oncology, surgical oncology, and genetics.

Challenging clinical scenarios were addressed through multidisciplinary debates and case reviews, including nuanced discussions on axillary surgery after neoadjuvant therapy, the management of lymphedema, and lobular breast cancer. Additionally, the programme provided updates on systemic therapy, central nervous system metastases, and novel treatment strategies shaping the future of breast cancer management. The symposium also celebrated emerging research by showcasing the best Canadian abstracts submitted this year, reinforcing the conference’s commitment to fostering innovation and collaboration. This conference report provides an overview of the themes and practice-changing insights presented at CBCS 2025, reflecting the dynamic landscape of breast cancer care in Canada.

## 2. Patient Voices—Pregnancy and Breast Cancer

The first presentations of the day were by two women who shared their personal journeys with breast cancer in the context of their own fertility and family planning. Their compelling and emotional accounts highlighted how no two journeys are alike; despite what these two women have in common, being diagnosed with breast cancer during their reproductive years, their experiences were very different.

### 2.1. Patient Voice I

“Privilege and luck”: this is how Jaclyn Greenberg summarizes her journey navigating breast cancer and fertility. In 2019, at the age of 34, she was diagnosed with hormone receptor-positive, human epidermal growth factor 2-negative (HR+/HER2-) invasive ductal carcinoma (IDC) stage 3b with lymph node involvement. She had just finished her master’s in health law and had spent years working with healthcare professionals, including in the context of clinical trials. She describes her medical knowledge, ability to evaluate, make informed decisions and advocate for her own health as privileges that many do not share. She says that when it came time to navigate her health journey, she drew on every ounce of her privilege.

Along with privilege, Jaclyn lists luck as the other driving force in her journey—luck in getting cancer-free, staying cancer-free, and ultimately, living her life with the family she wanted. That outcome took years of relentless treatment, decisions, and setbacks.

With a husband and toddler to think of, Jaclyn weighed how to be as aggressive as possible against cancer and how to preserve her fertility. She consulted an “army” of experts, from oncologists and researchers to bioethicists and fertility specialists, all of whom agreed it was reasonable to delay chemotherapy for fertility preservation (although none would overtly say that it was safe). Ultimately, she and her husband prioritized protecting the family they already had, and she embarked on her path through chemotherapy, surgery, radiation, and endocrine therapy.

When a doctor later told her that her ovaries were “quiet” and her hopes for fertility “futile,” she absorbed the blow while juggling radiation burns, a global pandemic, and a hungry toddler waiting for dinner. “I didn’t mourn the loss of my breasts,” she recalls. “I mourned the loss of my choices.” Still, she persisted. A second opinion and a negative ctDNA test in 2022 (as part of clinical trial screening) gave her permission to hope again. She asked herself: what would I do if I weren’t driven by fear? Within months of opting to take an endocrine therapy (ET) holiday, against all odds, she conceived naturally.

Today, Jaclyn is back on ET and enrolled in the CAMBRIA trial. Her son is 8; her “scientifically unexplainable” daughter is 2. Looking back, she urges doctors to help patients see the full circle of their present and future. “Bridge the privilege gap,” she asks, “so no one has to rely on luck the way I did”.

### 2.2. Patient Voice II

For Tabitha Holman, “missed chances and systemic failures” is how she describes her journey. A teacher, wife, and mother of two boys, she was 40 years old when she was diagnosed with HR-/HER2+, de novo metastatic breast cancer.

This devastating diagnosis on Thanksgiving weekend in 2023 marked the bitter culmination of a long struggle to be heard by medical professionals, which began in her 20s when a breast lump was dismissed as a harmless cyst because she was “too young” for breast cancer and had no family history.

Many years later, shortly after giving birth to her second child, Tabitha was in the process of navigating the healthcare system for an unrelated congenital heart condition. She raised concerns about a persistent lump in her breast that she noticed during breastfeeding and was told it was likely a blocked duct or hormonal changes. Google searches, medical reassurances, and a string of normal heart tests (including cardiothoracic imaging that she believed would have seen breast cancer) created a false sense of security.

When swollen lymph nodes drove her back to seek help, she faced a new barrier: clinics refusing mammograms because she did not meet high-risk criteria. She called 11 different clinics before she was accepted. Within minutes of that scan, everything had changed, as an emergency biopsy was ordered on the spot. And 3 weeks later, on Thanksgiving weekend, she received devastating news in the form of an impersonal, automated email from MyChart: the disease had spread before she even had a chance to fight it.

Over the next few months, Tabitha juggled fertility treatments for egg retrieval, financial fights for unfunded drugs, and painful procedures—all while parenting toddlers and preparing them for a future without her. She took family photos in the park the day before she started cancer treatment for the remainder of her life, she recorded bedtime stories, wrote cards for milestones she may never see, and wondered whether her youngest would remember her at all.

Just one week after Tabitha’s stage 4 diagnosis, Ontario lowered the screening age to 40—too late for her, but perhaps a chance for others. Tabitha reflects on the healthcare system that denied her timely screening and dismissed her symptoms until it was too late. “We get lost in mixed messaging and barriers,” she says. “I hope women don’t continue to fall through the same cracks, and that we keep moving closer to making metastatic breast cancer curable.”

## 3. Breast Cancer in 2025—Top Papers

The top papers for radiation therapy, surgical oncology, and systemic therapy are reviewed below and summarized in Table 1.

### 3.1. Radiation Therapy

#### 3.1.1. Europa [1]

With growing efforts to minimize treatment burden in older women with low-risk breast cancer, the phase 3 EUROPA trial compared radiotherapy (RT) and ET alone after breast-conserving surgery (BCS) in women aged 70+ with stage I, luminal A-like disease. In this interim analysis of 207 patients at 24 months, RT (mostly partial breast irradiation over 5 fractions) better preserved health-related quality of life (HRQOL) and resulted in fewer treatment-related adverse events (AEs) than ET (5–10 years of hormonal therapy). While these findings suggest RT may be a less burdensome option for some older patients, final results on local recurrence—the primary endpoint—are awaited. These results support RT as a promising option in carefully selected patients, pending long-term data. An outstanding question is the role of RT alone in the setting of an increasing trend toward omission of sentinel lymph node biopsy (SLNB).

#### 3.1.2. Hypog-01 [2]

As shorter radiation schedules gain traction to reduce treatment time and burden, the UNICANCER HypoG-01 phase 3 trial tested whether moderately hypofractionated (HF) locoregional radiation (40 Gy in 15 fractions over 3 weeks +/− boost at investigator’s discretion) after breast cancer surgery is as safe as normofractionation (NF; 50 Gy in 25 fractions over 5 weeks +/− boost) based on the 3-year cumulative incidence of arm lymphedema. The study evaluated 1221 patients. Lymphedema rates were high (~33%) in both groups, likely reflecting high rates of axillary surgery (>80%), which may be less common in current practice. Early toxicity at six months was slightly lower with HF-RT (45% vs. 52% grade ≥ 2 events), mainly for dermatitis, fatigue, and pain, with excellent cosmetic outcomes in both groups. This study demonstrates no safety concerns for HF nodal RT and supports the use of HF as a standard in patients receiving locoregional RT.

#### 3.1.3. Trog 20.03 Avatar [3]

With growing interest in delaying treatment changes for metastatic breast cancer, the AVATAR phase II trial tested whether stereotactic ablative body radiotherapy (SABR) to oligoprogressive sites could extend the duration of cyclin-dependent kinase 4 and 6 (CDK4/6) inhibitor plus aromatase inhibitor (AI) therapy in patients with estrogen receptor-positive (ER+), HER2− disease. In this multicenter, single-arm study of 32 patients, 47% remained event-free at 6 months, and 44% maintained systemic therapy without change for 12 months. Median progression-free survival (PFS) was 5.2 months, and modified PFS—which included additional SABR treatments—was 9.9 months. No grade 3+ toxicities were reported. These promising results suggest SABR could offer a well-tolerated strategy to safely extend the effectiveness of current systemic therapies in carefully selected patients.

### 3.2. Surgical Oncology

#### 3.2.1. Risk Reducing Surgeries in BRCA1/2 Mutation Carriers: More Is More [4]

In young patients with breast cancer who carry a germline pathogenic variant of *BRCA1* or *BRCA2* (g*BRCA1/2^mut^*), the survival impact of risk-reducing surgeries remains unclear. This large, international cohort study examined whether risk-reducing mastectomy (RRM) and/or risk-reducing salpingo-oophorectomy (RRSO) improved survival in women diagnosed with stage I–III breast cancer at age 40 or younger. Among 5290 patients followed for a median of 8.2 years, both surgeries were independently associated with better overall survival (OS): RRM [adjusted (hazard ratio) HR 0.65) and RRSO (adjusted HR 0.58), regardless of tumour subtype or nodal status. These findings suggest that risk-reducing surgeries may meaningfully improve long-term survival in young *BRCA* carriers, offering important evidence to guide individualized risk-reduction discussions in this high-risk population.

#### 3.2.2. Axillary Surgery in Breast Cancer—Insema: Less Is More [5]

With growing focus on minimizing surgical interventions in early breast cancer, the INSEMA phase 3 trial evaluated whether omitting SLNB compromises outcomes in patients with clinically node-negative, T1–T2 breast cancer undergoing BCS. In this large, randomized trial of 4858 patients, omission of SLNB was noninferior to SLNB for invasive disease-free survival (DFS) at 5 years (91.9% vs. 91.7%; HR 0.91), with slightly higher axillary recurrence (1.0% vs. 0.3%) but lower rates of lymphedema, better arm mobility, and less pain. These findings suggest that SLNB may be safely omitted in select low-risk patients—particularly postmenopausal women with ER+/HER2−, clinically node-negative disease—supporting a shift toward less invasive axillary management in appropriately selected cases.

#### 3.2.3. Active Monitoring for Low-Risk DCIS—Comet: Less Is the Same [6]

Given concerns about overtreatment of low-risk ductal carcinoma in situ (DCIS), the COMET phase 3 trial evaluated whether active monitoring could safely replace surgery for women with HR+, grade 1–2 DCIS. In this randomized study of 957 patients, the 2-year risk of ipsilateral invasive cancer was 4.2% with active monitoring versus 5.9% with standard surgery (with or without radiation), meeting the noninferiority threshold. Invasive cancer characteristics were similar between groups, and most patients in the active monitoring arm avoided surgery. While these early results suggest that active monitoring may offer a safe short-term alternative to surgery for select patients with low-risk DCIS, longer follow-up is needed before changing practice, with ongoing efforts focused on identifying biomarkers to better predict progression.

### 3.3. Systemic Therapy of Breast Cancer

Rather than highlighting individual studies, the speaker reframed the session to spotlight three pressing therapeutic challenges, each shaped by multiple pivotal trials.

#### 3.3.1. Mutational Mayhem in ER+, HER2-Low Metastatic Breast Cancer: Targeting PIK3CA/AKT, ESR1

Mutations in PI3K, AKT, and particularly *ESR1* are now recognized as common drivers of resistance in ER+/HER2− metastatic breast cancer. While CDK4/6 inhibitors plus ET remain standard first-line treatment, disease progression is inevitable, and a new wave of mutation-targeted strategies is reshaping care. The SERENA-6 trial showed that detecting *ESR1* mutations before progression and switching to the oral selective endocrine receptor degrader (SERD), camizestrant, led to a 7-month PFS gain [7]. INAVO-120 demonstrated that PI3K inhibition with inavolisib in early relapsed disease—paired with palbociclib + fulvestrant—yielded significant OS and PFS benefits in aggressive disease [8]. In second-line, FINER and VERITAC-2 targeted AKT and ER degradation, respectively, with each trial demonstrating benefit in mutation-selected patients [9,10]. EMBER-3, also a second-line trial, demonstrated significant PFS benefits of abemaciclib + imlunestrant (oral SERD) over imlunestrant alone, regardless of *ESR1* mutation [11]. Exploratory data from DESTINY-Breast06 also confirmed that trastuzumab deruxtecan (T-DXd) retains efficacy across mutational subtypes, including PI3K, *ESR1* and *BRCA* [12]. The key challenge now lies not in identifying targets, but in ensuring timely, standardized biomarker testing and balancing efficacy with real-world tolerability—especially as newer agents bring unique toxicity profiles.

#### 3.3.2. Whats’s New with Old Foes—TNBC and HER2+: Doubling (Tripling) Down on Multiple Targets

Combination strategies are reshaping first-line treatment for metastatic triple negative breast cancer (TNBC) and HER2+ breast cancer. In programmed death-ligand 1-positive (PD-L1+) metastatic TNBC, the ASCENT-04 trial showed that replacing chemotherapy with the antibody-drug conjugate (ADC), sacituzumab govitecan (SG), plus pembrolizumab significantly improved PFS—an important advance given that up to half of patients never reach second-line therapy [13]. In metastatic HER2+ disease, DESTINY-Breast09 demonstrated that T-DXd combined with pertuzumab outperformed the standard taxane-trastuzumab-pertuzumab (THP) regimen, likely setting a new first-line standard [14]. Results from the T-DXd monotherapy arm are still pending. Meanwhile, the PATINA trial found that adding palbociclib to maintenance HER2-targeted and ET in ER+/HER2+ metastatic disease extended PFS, supporting the value of multi-targeted regimens [15]. These findings highlight a growing trend toward dual- and triple-agent strategies in the metastatic setting. However, their real-world applicability remains uncertain, as many trial participants had not received prior adjuvant pembrolizumab or trastuzumab emtansine (T-DM1). As always, translating these advances into practice will require thoughtful consideration of toxicity, treatment history, and individual patient factors.

#### 3.3.3. Behind Enemy Lines: Brain Metastases—Some More Promise

Brain metastases remain one of the most challenging frontiers in metastatic breast cancer, particularly for HER2+ disease. DESTINY-Breast12 offered renewed hope, showing that patients with HER2+ brain metastases treated with T-DXd achieved a striking 90% overall survival at 12 months—an impressive outcome in a historically difficult-to-treat population. While this is not the first evidence of ADC demonstrating central nervous system (CNS) activity, the HER2+ setting shows the most compelling results to date [16]. These findings reinforce the potential of ADCs like T-DXd—and possibly SG—to penetrate the CNS and offer meaningful control. Yet, choosing therapies based on intracranial vs. systemic progression remains nuanced, and real-world sequencing continues to be a clinical challenge.

## 4. Keynote Address

### Global Health/Breast Cancer

This keynote presentation highlighted widening disparities in breast cancer outcomes between high-income and low- and middle-income countries (LMICs), while outlining scalable solutions to improve equity worldwide. Breast cancer is now the most common cancer among women globally, including in many low-resource settings where cases are rising sharply [17]. While mortality continues to decline in high-income countries due to early detection and improved treatments, many women in LMICs face late-stage diagnoses, limited treatment access, and broader social impacts—such as leaving behind children who are “cancer orphans”—underscoring the urgent need for equitable care models that reflect local realities [18]. As Dr. Verna Vanderpuye (Korle Bu Teaching Hospital, Accra, Ghana, Africa) poignantly once said, “You have to understand me to help me”—a reminder that many well-intentioned solutions often fail by overlooking the social and cultural realities shaping care in these LMIC settings.

The talk featured several innovative, real-world projects addressing these challenges. In Bangladesh, a breast cancer clinic empowered local women through education, navigation support, and mobile technology, improving early detection [19]. In India, community health worker-led clinical breast exams successfully detected early-stage cancers and reduced mortality in older women [20]. These, and other examples informed the WHO’s Global Breast Cancer Initiative, which sets clear targets: increasing early-stage diagnosis rates, ensuring timely diagnosis within 60 days, and improving treatment completion rates [21].

The speaker emphasized that achieving breast cancer equity will require more than medical advances alone. Solutions must address social and economic barriers—such as stigma, misinformation, financial hardship, and prioritize patient-centred, community-driven programmes like navigation services and decentralized care models. The central message was clear: meaningful progress in breast cancer care worldwide depends not only on scientific innovation but also on sustained efforts to dismantle systemic barriers to care.

## 5. Breast Cancer and Systemic Therapy Updates

Systemic therapy updates for breast cancer were presented for hormone receptor-positive (early and metastatic), HER2+, and TNBC subtypes. The section summary can be found in Box 1.

Box 1 **Section 5 summary.**

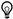
 Section takeaways

- In early ER+ disease CDK4/6 inhibitors have redefined adjuvant care, oral SERDs are advancing into the early setting, ctDNA testing for minimal residual disease is promising, and the future direction is shifting toward tailored, biomarker-driven treatment.
- In metastatic ER+ disease, sequencing of CDK4/6 inhibitors is still an outstanding question, next-generation oral SERDs are promising options, the inavolisib triplet has shown OS benefit as PI3K-directed therapy, and ADC are being used as new options for later lines of therapy.
- In HER2+ disease, T-DXd is redefining initial management, efficacy of T-DXd has been established for brain metastases, and earlier use is under investigation. The PATINA regimen has shown benefit for triple-positive metastatic disease.
- In triple-negative disease, ADCs are transforming the landscape and offering chemo-free options in later lines of treatment. ADCs are also moving earlier in treatment, but immunotherapy remains essential for PD-L1+ disease.

### 5.1. Advances in ER+ Breast Cancer

#### 5.1.1. Early Setting

Recent results from the monarchE and NATALEE trials continue to reshape early ER+ breast cancer treatment, placing CDK4/6 inhibitors at the centre of adjuvant care. Both trials confirmed consistent benefits with abemaciclib and ribociclib, respectively, in high-risk stage II–III disease, firmly establishing their role—though each comes with trade-offs [22,23]. Abemaciclib, given continuously, shows sustained benefit but carries notable gastrointestinal side effects. However, new dose-escalation strategies—starting at lower doses and gradually increasing—have shown promise in improving tolerability and reducing early discontinuation, helping more patients stay on therapy [24].

With CDK4/6 inhibitors now well-established, the next frontier involves reducing chemotherapy use. Trials testing CDK4/6 inhibitors as potential chemotherapy alternatives are underway, though longer follow-up is needed to confirm this approach. At the same time, efforts to refine chemotherapy decisions are progressing. Biomarkers such as anti-Müllerian hormone (AMH) may help identify premenopausal women who could safely avoid chemotherapy when treated with ovarian suppression and ET. This aligns with the major 15-year SOFT and TEXT update, which firmly reinforced the long-term benefit of combining ovarian function suppression (OFS) with AI, especially in women under 35 or with aggressive tumours [25]. These results cemented OFS plus AI as a key standard in high-risk premenopausal patients. However, this longstanding ET standard may soon evolve. The growing enthusiasm for oral SERDs in metastatic breast cancer is now extending into the early-stage setting. These agents, already showing effectiveness in advanced disease, are being actively studied as potential alternatives to AI in early breast cancer—raising the possibility that they could eventually reshape the ET backbone in selected patients.

Meanwhile, other innovations are advancing. CtDNA testing is being explored to detect minimal residual disease (MRD) and guide treatment, though its clinical role remains investigational. Immunotherapy also remains exploratory, with early trials such as KEYNOTE-756 and CheckMate-7FL suggesting potential benefit in high-risk, node-positive, PD-L1+ subgroups, but current data remain immature [26,27]. Together, these shifts point to a rapidly evolving treatment landscape, where chemotherapy, ET, and targeted therapy are all being redefined.

#### 5.1.2. Metastatic Setting

CDK4/6 inhibitors remain the cornerstone of first-line treatment in metastatic ER+ breast cancer. However, whether they can be re-used after progression remains uncertain. Trials like MAINTAIN and Post-Monarch suggest that switching both the CDK4/6 inhibitor and endocrine partner may modestly improve PFS, but benefits remain limited, and there is no clear consensus yet on second-line CDK4/6 use [28,29].

Meanwhile, oral SERDs—easier to take than intramuscular fulvestrant—are gaining traction in the second-line setting for *ESR1*-mutant tumours. Trials like EMERALD (elacestrant), SERENA-2 (camizestrant), and EMBER-3 (imlunestrant) have shown benefit, leading to approvals in select patients [11,30,31]. The first PROTAC degrader, vepdegestrant (VERITAC-2), is in development and may offer better tolerability than SERDs, though more data are needed [32]. Among these studies, SERENA-6 has sparked debate by targeting molecular progression (detected via ctDNA) before clinical progression—a strategy that improved PFS but raises questions about its broader use [7].

A major recent advance is the INAVO120 triplet—inavolisib plus fulvestrant and palbociclib—for *PIK3CA*-mutant endocrine-resistant disease. It showed striking PFS and the first-ever OS benefit for a PI3K-targeted therapy (27 vs. 34 months), though side effects like hyperglycemia require monitoring [33]. The ongoing INAVO123 trial is testing whether this triplet can be adapted to endocrine-sensitive patients using AI instead of fulvestrant. Similarly, the Canadian-led MA.40/FINER trial confirmed AKT inhibition (ipatasertib) as an option for AKT-altered tumours, reinforcing the growing role of ctDNA-guided precision therapy [9]. The PI3K pathway field is also moving toward mutant-selective allosteric inhibitors (e.g., RLY-2608, STX-478, OKI-219) that may avoid the toxicities seen with earlier drugs.

In later lines of treatment, ADCs have transformed treatment for endocrine-refractory disease. T-DXd has shown strong efficacy in HER2-low and -ultra-low tumours, improving PFS and OS in heavily pretreated patients and recently in chemo-naive populations as well [34,35]. SG and datopotamab deruxtecan (data-DXd) offer additional options in later lines, though their benefits are more modest, and treatment sequencing remains unclear [36,37,38]. Trials are ongoing to determine optimal ADC sequencing, as real-world data suggest mixed outcomes after prior ADC exposure.

Overall, metastatic ER+ breast cancer therapy is increasingly guided by molecular profiling, with expanding targeted options in earlier lines—but challenges around sequencing, tolerability, and long-term outcomes remain.

### 5.2. Advances in HER2+ Breast Cancer

HER2+ breast cancer treatment is evolving rapidly, driven by T-DXd. The DESTINY-Breast09 trial marked a major shift in the first-line metastatic setting, showing that T-DXd plus pertuzumab significantly outperforms standard THP chemotherapy in PFS [14]. While this approach brings longer treatment durations and some risk of pneumonitis, most cases are low-grade and can be managed with careful monitoring and, in some cases, safe rechallenge. The ongoing DEMETHER trial is also exploring shorter T-DXd induction followed by HER2-targeted maintenance, aiming for a more tolerable long-term strategy [39].

T-DXd’s impact is not limited to first-line treatment. The DESTINY-Breast12 trial showed impressive results in patients with brain metastases, with high intracranial response rates and durable control [16]. This reinforces its key role in Canadian treatment guidelines, where T-DXd is already prioritized for patients with brain metastases [40]. Meanwhile, in early-stage disease, the DESTINY-Breast05 trial is testing T-DXd as adjuvant therapy for patients with residual disease, potentially extending its reach into curative settings [41].

Alongside these shifts, HER2+/HR+ (triple-positive) metastatic breast cancer treatment is also evolving. The PATINA trial demonstrated that adding a CDK4/6 inhibitor (palbociclib) to HER2-targeted maintenance therapy with ET can improve PFS, supporting more personalized approaches for these patients [15]. Chemo-free CDK4/6 regimens are also being explored for those who may not tolerate traditional treatment.

### 5.3. Advances in TNBC

Chemotherapy has been the backbone of TNBC treatment for decades—but despite additions like pembrolizumab for PD-L1+ metastatic disease (KEYNOTE-355), outcomes remain poor, with survival often measured in months. Now, a new generation of therapies—ADCs—is shifting the treatment landscape. ADCs are proving not only to be effective in heavily pretreated metastatic TNBC but are beginning to challenge chemotherapy in earlier lines of therapy. This shift began with the pivotal ASCENT trial, where SG significantly improved both PFS and OS versus chemotherapy in heavily pretreated metastatic TNBC [42]. SG became a new standard in later lines, showing consistent benefit across subgroups, including patients with low HER2 expression, and regardless of trophoblast cell surface antigen 2 (TROP-2) levels. Neutropenia remains the primary toxicity concern but is manageable with proactive strategies.

Building on ASCENT, ASCENT-04/KEYNOTE-D19 delivered practice-changing results by showing that SG combined with pembrolizumab significantly improves first-line PFS in PD-L1+ metastatic TNBC, effectively replacing chemotherapy in this setting [13]. Although OS data are still maturing, this combination has quickly become a new first-line standard for eligible patients. Trials in PD-L1-negative metastatic TNBC such as SG in first-line (ASCENT-03), will be reported soon.

Beyond metastatic disease, ADCs are being pushed into curative-intent settings. Several adjuvant trials—including OptimICE-RD (NCT05633654), TROFUSE-12 (NCT06393374), and TROPION-Breast03 (NCT05629585)—are testing ADCs (with or without immunotherapy) for patients with residual disease after neoadjuvant therapy [43,44,45]. At the same time, neoadjuvant trials like TROPION-Breast04 and TROFUSE-032 (NCT06966700) are evaluating ADC-based regimens head-to-head against established chemo-immunotherapy backbones such as KEYNOTE-522 [46,47,48]. Collectively, these trials show that ADCs are already reshaping TNBC treatment offering powerful new options where few existed before. Yet, as the presenter emphasized, their full potential will come with better biomarkers to guide treatment and reduce toxicity. With ongoing work on patient-derived models and deeper immune profiling, there is strong hope that future treatment strategies will not only be more effective, but also more precisely tailored to each patient.

## 6. Debate: Is ctDNA Ready for Prime Time in the Management of Early Breast Cancer

Presenters offered contrasting viewpoints regarding the use of ctDNA in early breast cancer. A summary appears in Table 2.

### 6.1. No

While ctDNA has strong prognostic power, ctDNA is not ready to guide treatment decisions in early-stage breast cancer. Unlike metastatic disease—where actionable mutations can inform therapy—early breast cancer lacks validated ctDNA-driven targets. ctDNA levels are often too low to detect reliably, and current assays cannot distinguish whether positivity reflects subclinical metastatic disease or loco-regional persistence. Most importantly, no trial has yet shown that intervening based on ctDNA improves survival, and guidelines from major oncology societies do not recommend ctDNA monitoring outside research settings [49,50,51]. Without clarity on whether, when, or how to act on positive results, ctDNA risks causing anxiety without actionable benefit. The field still requires prospective studies to define optimal timing, frequency, thresholds for intervention, and whether ctDNA-triggered therapies can change outcomes.

### 6.2. Yes

CtDNA testing was presented as ready for selective application in early breast cancer, particularly in cases where treatment decisions remain uncertain. This point was illustrated through the case of a young, high-risk patient who had completed standard therapy but continued to face unresolved questions regarding her true risk of recurrence and whether additional treatment was warranted. Such scenarios reflect a common clinical dilemma: patients often ask how they can be certain their cancer is truly gone. The rationale for ctDNA lies in its potential to address this gap. Modern assays now demonstrate high sensitivity and reproducibility, particularly when serially measured. Multiple studies show that detectable ctDNA strongly predicts recurrence, often providing lead times of 7–27 months before clinical relapse [22,52]. In contrast, persistently negative ctDNA offers substantial reassurance, with very low recurrence rates observed in serial surveillance cohorts. These features support ctDNA’s role in risk stratification, shared decision-making, and easing patient concerns about recurrence, future fertility, and long-term planning. In carefully selected cases—such as high-risk patients with unresolved questions after standard therapy—ctDNA may help clarify prognosis and align treatment intensity with individual risk. While not yet a universal standard, ctDNA’s ability to personalize survivorship care and reduce overtreatment represents a meaningful, real-world benefit.

## 7. The Latest Advances in…

This section covered the latest advances in radiology and screening, surgical oncology, and genetics. The section summary appears in Box 2.

Box 2 **Section 7 summary.**

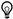
 Section takeaways

- Expanding germline testing and integrating early results into care can guide preventive strategies, optimize systemic therapy, and enable cascade testing to reduce cancer risk in families.
- Modern trials support safe de-escalation of radiotherapy through shorter regimens, omission in selected low-risk patients, and re-evaluation of nodal radiation.
- Breast cancer screening strategies must evolve to address biologic aggressiveness in younger and minority women, integrate supplemental imaging and artificial intelligence, and close equity gaps in access and outcomes.
- Emerging trial data suggests that omission of breast or axillary surgery may be feasible in carefully selected patients after neoadjuvant therapy.

### 7.1. Radiology and Breast Screening

Mammography remains the gold standard for breast cancer screening, with evidence showing it can reduce mortality ranging from 27–59%, depending on age and province [53]. However, it also has important limitations, as it may over-detect indolent cancers and miss aggressive disease. These challenges are especially relevant among younger women and racial and ethnic minority groups, who often present with more aggressive disease and for whom current screening strategies may be less effective.

Breast cancer is the leading cause of cancer death in women under 40, who more often present with aggressive disease. While studies have shown that women in their 40s benefit from screening [54], the Canadian Task Force for Preventive Health recommends against screening before age 50, a stance not widely shared by patient advocacy groups and healthcare practitioners.

Beyond age, racial and ethnic background also plays a critical role in shaping breast cancer risk and outcomes. Black, Hispanic, and Asian women have higher rates of triple-negative disease and peak incidence in their 40s, compared to the 60s in white women. Minority women are 72% more likely to be diagnosed with invasive breast cancer before age 50 and 127% more likely to die from it, independent of socioeconomic status [55].

Supplemental imaging can improve detection in women with some of the aforementioned risk factors. Magnetic resonance imaging (MRI) has been shown to be the most reliable supplemental screening modality, increasing cancer detection rate by 1.52/1000 in a meta-analysis [56], with abbreviated MRI performing similarly to full MRI [57]. Contrast-enhanced mammography performs similarly to MRI in both high-risk and dense-breast average-risk populations [58].

Screening disparities disproportionately affect minorities and other underserved groups. To prevent widening gaps in breast cancer outcomes, equitable adoption of advanced imaging and updated guidelines are essential.

### 7.2. Radiation Oncology

Breast radiotherapy aims to reduce local recurrence, maximize breast preservation, and improve DFS, all while minimizing both patient and system burden. Large trials previously established hypofractionation, consisting of 40Gy in 15 fractions (40Gy/15) delivered over three weeks, as a global standard [59,60]. Recent advances have focused on further reducing treatment time and omitting radiation in select patients.

The UK FAST-FORWARD trial compared standard hypofractionation to two ultra-hypofractionated one-week regimens, 26Gy/5 and 27Gy/5, in predominantly low-risk patients undergoing BCS. Ten-year results confirmed similar results between the 26Gy/5 and 40Gy/15 regimens, indicating that ultra-hypofractionation resulted in non-inferior local control [61].

Omission of breast RT in low-risk ER+/HER2− patients on ET has been supported by trials such as PRIME II, which reported a 10-year local recurrence of 9.5% without RT versus 1% with it [62]. Other studies have used biomarker criteria, such as Ki-67 or Oncotype DX Recurrence Score, to define groups of patients with a 1–2% five-year recurrence risk [63,64].

In node-positive disease, regional nodal radiation has long been standard, but its necessity is being reassessed in the context of modern systemic therapy. The NSABP B-51/RTOG 1304 trial examined omission of nodal radiation in patients who were initially node-positive but became node-negative after neoadjuvant therapy. At five years, omission did not increase recurrence or compromise survival [65].

These developments collectively indicate a trend toward de-escalating RT in appropriately selected patients, reducing treatment time and toxicity without compromising outcomes.

### 7.3. Surgical Oncology

Recent research in surgical oncology has increasingly focused on safely reducing the extent of surgery without compromising outcomes. One such area of investigation is the omission of breast surgery after neoadjuvant therapy in patients with complete clinical response. A single-arm, multicentre study enrolled patients with low-risk breast cancer who, after neoadjuvant chemotherapy, had less than 2 cm of residual abnormality on imaging and negative tumour-bed biopsy. At a median follow-up of 55.4 months, there were no ipsilateral breast tumour recurrences [66,67].

In contrast, the Canadian-led NRG-BR005 trial tested tumour-bed biopsy as a predictor of pathologic complete response, followed by standard surgery in all cases. Among 101 evaluable patients, the negative predictive value was only 78.3%, indicating insufficient sensitivity for reliably selecting patients for omission of surgery [68]. Other studies have also reported high false-negative rates, though subgroup analyses suggest that low residual imaging abnormality (<2 cm) and ≥6 vacuum-assisted biopsies may reduce false negatives [69].

Another focus is axillary management in patients with residual node-positive disease after neoadjuvant chemotherapy. While guidelines still recommend axillary lymph node dissection (ALND), ongoing trials are investigating whether axillary radiation alone may suffice. Supporting this approach, a Turkish registry study reported a 0.3% recurrence rate in patients managed without completion axillary dissection, equivalent outcomes to those who became node-negative following neoadjuvant chemotherapy [70]. Similarly, a secondary analysis of the I-SPY-2 trial likewise found no significant difference in recurrence or survival between SLNB alone and biopsy with subsequent ALND [71].

### 7.4. Genetics

In Ontario, most publicly funded genetic testing for breast cancer remains criteria-based, relying on personal and family history. By contrast, mainstream or oncology-initiated testing of newly diagnosed breast cancer patients can reduce missed cases and improve equity, though not all patients are suitable candidates. A recent Canadian guideline adaptation from the ASCO recommendations supports testing all patients aged 65 years or younger with invasive breast cancer or DCIS, as well as select patients over 65 based on tumour subtype or a strong family history [72].

For *BRCA1/2* carriers, the risk of contralateral breast cancer is high, and contralateral risk-reducing mastectomy (CRRM) is highly effective in prevention. However, evidence for a survival benefit is mixed: some large cohort studies have demonstrated significant reductions in mortality [4], while others have shown no survival advantage [73]. Young patients with high lifetime risk are most likely to benefit from CRRM, and decisions should account for non-survival factors, such as the burden of surveillance.

The OlympiA trial demonstrated that one year of adjuvant olaparib in *BRCA1/2* carriers with residual disease after neoadjuvant therapy yields sustained improvements in OS at six years [74]. These results underscore the importance of early genetic testing to identify eligible patients.

Despite its benefits, cascade testing uptake among relatives of mutation carriers remains suboptimal, largely due to reliance on the proband to communicate results. Alternative strategies include traceback testing, digital tools for sharing family pedigrees and results, and practical measures such as providing patients with referral forms for relatives. Incorporating family testing reminders into oncology practice can help prevent cancers in at-risk relatives.

## 8. Challenging Clinical Cases with a Debate

The session began with a debate on whether patients with one positive residual lymph node after neoadjuvant therapy require a full ALND followed by a discussion on a patient case. Section summary appears in Box 3.

Box 3 **Section 8 summary.**

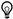
 Section takeaways

- Patient-specific factors often outweigh rigid adherence to treatment guidelines, underscoring the need for individualized decision-making.

### 8.1. Yes

Patients who remain node-positive following neoadjuvant treatment tend to have more aggressive tumour biology, reflecting a chemoresistant disease state, particularly in triple-negative and HER2+ subtypes. Historical data indicate that these patients are at high risk of recurrence, with 10-year loco-regional recurrence rate of 16–22% [75]. Observational series suggest that those with residual micrometastatic or macrometastatic nodal disease post-neoadjuvant therapy frequently have additional positive nodes not detected by SLNB, making omission of further axillary surgery potentially unsafe [76,77]. Available studies, while sometimes reassuring, are limited by selection bias, short follow-up, and higher recurrence rates in high-risk subgroups, especially TNBC. Thus, current guidelines are justified in recommending continued use of ALND in such cases until stronger level-one evidence supports de-escalation.

### 8.2. No

The necessity of full ALND in patients with minimal nodal involvement after neoadjuvant therapy is increasingly questioned. Large historical trials have shown no significant difference in outcomes between node dissection and axillary radiation for node-positive disease [78,79,80]. Recent data reveal that 67% of patients with one positive sentinel node do not harbour additional nodal disease, meaning they would not benefit from completion dissection and could instead suffer harm from surgical morbidity [81]. Furthermore, changes in adjuvant therapy choices are rarely dictated by exact nodal count, reducing the value of further axillary surgery for staging purposes alone. Ongoing and upcoming trials are expected to clarify these issues, but a move toward joint clinician-patient decision-making, considering individualized risks and benefits, was emphasized.

### 8.3. Multidisciplinary Case Discussions—Management of Patients with Stage IV Disease

The first case discussion focused on a premenopausal woman with de novo stage IV, HR+/HER2- breast cancer, with a T2 primary tumour (4.2 cm on imaging). Staging revealed skeletal metastatic disease in the sternal body. Treatment was initiated with AI + CDK4/6i resulting in stable bone metastasis but the patient experienced increasing local symptoms from her breast mass. The presenter discussed the merits of palliative surgery, palliative radiation, or systemic therapy. While surgery offers symptom relief, it carries risks and does not confer oncologic or survival benefit. Treatment should be tailored to symptom control, patient preferences, and multidisciplinary input, and local therapy for metastatic disease should not be pursued solely for survival improvement.

The second case was a 46-year-old premenopausal woman presenting with a palpable left central breast mass measuring 4 × 6 cm and palpable left axillary nodes. Core biopsy revealed IDC, grade 2, ER/PR−/HER2+ [3+ on immunohistochemistry (IHC)]. Fine-needle aspiration of the axilla confirmed metastatic mammary carcinoma. Breast MRI demonstrated a 4.5 × 4.3 × 6.0 cm central mass with multiple enlarged axillary nodes and two enlarged internal mammary nodes. Positron emission tomography (PET) and computer tomography (CT) identified single liver metastasis confirmed by biopsy. Discussion centred around whether curative intent is appropriate in such oligometastatic presentations. Practices vary by region and access to imaging; some experts and institutions treat aggressively with multimodal therapy, aiming for no evidence of disease, while others maintain a palliative approach due to lack of supporting randomized data. Considerations include disease subtype, patient age, stability on current therapy, and multidisciplinary consensus.

Multidisciplinary review and shared decision-making are critical elements of treatment planning. Heterogeneity in practice, even within multidisciplinary tumour boards, leads to variability in care and patient access to aggressive treatments.

## 9. Surgical Symposium: Management of Lymphedema

The session focused on the surgical management of lymphedema with a summary appearing in Box 4.

Box 4 **Section 9 summary.**

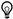
 Section takeaways

- ILR with lymphovenous bypass can be used to reduce lymphedema risk by rerouting lymphatics during ALND. This method requires microsurgery expertise and specialized equipment.
- S-LYMPHA is an alternative to ILR that reconnect lymphatics to nearby veins using simple tools. This technique is more accessible and feasible in centres without microsurgical expertise.
- Structured surveillance protocols (pre-op to post-op) reduce lymphedema rates with early-stage cases being reversible. Tools like risk calculators are refining prevention strategies.

### 9.1. Immediate Lymphatic Reconstruction with Lymphovenous Bypass at the Time of ALND

Lymphedema remains one of the most feared complications of ALND, affecting up to one in four breast cancer patients [82]. Once it develops, there is no definitive cure—treatments are limited, and quality of life (QoL) often suffers significantly [83,84]. This makes prevention crucial. One of the most promising preventive approaches is immediate lymphatic reconstruction (ILR) with lymphovenous bypass (also known as lymphatic microsurgical preventative healing approach [LYMPHA]), a plastic surgery technique that originated in Italy in 2009 and has since gradually gained momentum worldwide. It involves rerouting lymphatic channels to nearby veins during ALND [85]. Aided by a blue dye injection to the upper arm to help visualize lymphatic channels, this technique aims to restore drainage of the channels before swelling can develop.

In Canada, the speaker presented outcome data from a single centre ILR programme, reporting encouraging results with substantially lower lymphedema rates—around 8%—compared to historical rates of 25–30% [86]. Similar outcomes have been reported internationally, including in the only randomized controlled trial to date from Memorial Sloan Kettering, which also demonstrated similar reductions in lymphedema risk [87]. Still, ILR is not unanimously accepted as at least one real-world study has found no clear advantage over standard care [88]. Despite this, ILR is increasingly offered given its low surgical risk and high patient interest. It does require specific expertise, extra surgical time and seamless coordination between breast and plastic surgeons—an added logistical challenge. Nonetheless, the speaker strongly recommended offering ILR to all patients undergoing ALND, viewing it as a practical and effective step to help prevent debilitating lymphedema.

### 9.2. The S-Lympha Procedure

While ILR is highly effective, its need for specialized microsurgical skills and equipment limits its availability in many centres. One alternative approach is the Axillary Reverse Mapping (ARM) technique, where surgeons attempt to preserve arm-draining lymphatics during ALND. However, ARM does not restore lymphatic flow, and its effectiveness diminishes when cancer-involved nodes overlap with these lymphatics, necessitating their removal regardless of mapping accuracy [89,90].

To address these limitations, Simplified LYMPHA (S-LYMPHA) offers a middle ground combining the accessibility of ARM with the reconstructive intent of ILR. Rather than stopping at preservation, S-LYMPHA allows surgeons to actively reroute divided lymphatics by anastomosing them into nearby veins, typically the thoracic epigastric vein. This is done without a microscope using a “sleeve” insertion technique and basic sutures, relying on the vein’s low-pressure system to support lymphatic drainage. Because it requires only standard instruments and can be performed with loupes or even by direct vision, S-LYMPHA is both technically feasible and scalable, making lymphatic reconstruction accessible to a broader range of surgical practices and patients [91]. While S-LYMPHA may not match ILR’s precision, it offers a practical, low-barrier option for many centres—allowing breast surgeons to play a direct role in reducing lymphedema risk. Patient awareness and education on risk-reducing strategies are also important components to improve outcomes.

### 9.3. Towards Prospective Surveillance and Preventive Management of Lymphedema in High-Risk Individuals

Although surgical techniques like ILR and S-LYMPHA help reduce lymphedema following ALND, the condition remains a clinically significant complication, particularly in patients receiving axillary radiation or extensive nodal treatment [92]. Importantly, if detected early—before fibrosis develops—lymphedema is often reversible [93]. This underscores the value of proactive surveillance. A meta-analysis confirmed that structured protocols starting before surgery and continuing through follow-up can significantly reduce lymphedema rates [94]. As such, current guidance from the Multinational Association of Supportive Care in Cancer (MASCC) guidelines recommend baseline limb measurements before surgery, with follow-up every 3–4 months in year one and every 6–12 months after [95]. Most early cases are successfully managed with a class II compression sleeve worn for 1–6 weeks, preventing progression. If swelling persists, complex decongestive therapy may be added.

To further refine prevention, a Canadian team developed a risk prediction formula incorporating age, body mass index (BMI), breast density, ALND status, and the number of positive nodes [96,97]. This validated tool, helps objectively stratify patients and guide decisions around surveillance intensity and use of prophylactic interventions [96]. Beyond clinical risk factors, ongoing research through the DRAIN initiative at the Princess Magaret Cancer Centre in Toronto, is exploring cell-free DNA from surgical drain fluid as a biomarker for predicting both cancer recurrence and treatment-related toxicities like lymphedema, building on results showing feasibility and promising sensitivity compared to standard blood samples [98].

While surgical prevention is evolving, early detection and structured non-surgical surveillance remain the most practical and scalable strategy. The next challenge lies in implementing these approaches consistently across clinical settings.

## 10. Optimization Strategies for Lobular Breast Cancer

The session focused on lobular cancer, highlighting a patient’s lived experience as well as systemic treatment, surgical considerations, and research. The section summary appears in Box 5.

Box 5 **Section 10 summary.**

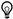
 Section takeaways

- ILC is a common breast cancer subtype with clinical and pathological features dis-tinct from IDC. The linear growth pattern in ILC resulting from the loss of E-cadherin can be difficult to detect on routine mammograms, often leading to delayed diagnosis.
- Some evidence in lobular disease supports anthracycline-containing chemotherapy in high-risk N2/N3 patients, ET with an AI (letrozole/anastrozole) in the adjuvant set-ting, and CDK4/6 inhibitors in the metastatic setting.
- MRI for surgical planning and PET/CT for staging distant disease are important considerations in lobular breast cancer. Principles of surgical resection in ILC remain the same as those for IDC, both within the breast and the axilla. Neoadjuvant chemotherapy for surgical downgrading may have a role in selected patients.
- Emerging research priorities in lobular breast cancer aim to standardize diagnosis, advance imaging techniques for accurate detection and staging, identify better gene signatures as predictors for outcomes in ILC, and provide evidence-based treatment options for patients with lobular breast cancer.

### 10.1. The Patient Voice—Living with Lobular Breast Cancer

Brenda Cunnnigton shared her story of living with invasive lobular carcinoma (ILC) and the years of missed opportunities for earlier detection with this “quiet” and “sneaky” cancer. ILC is clinically and pathologically distinct from invasive IDC, with a single-file growth pattern that is difficult to detect with routine mammogram and diagnosis often delayed until later stages [99].

It took five years after a lump was discovered in Brenda’s left breast before she was finally diagnosed with ILC in 2022. At the time she was 43 and enjoying her life to the fullest with her two children. In 2017, her initial two mammograms and ultrasound findings revealed heterogeneously and bilaterally dense breast parenchyma and multiple cysts in her left breast that were considered benign [Breast Imaging Reporting and Data System-2 (BI-RADS 2)]. A follow-up mammogram a year later showed a poorly defined area of increased vascularity within the left breast and a suspicious abnormality the surgeon confirmed was a simple cyst. For the next three years Brenda repeated the same tests on breast tissue considered dense enough to obscure pathology. She said, *“Had I known then, what I know now, I would have asked why. Why don’t we do a biopsy of the cyst? Can my dense breast tissue be hiding other areas of concern? Could another modality like a breast MRI be a better follow-up option at this point?”—Brenda Cunnington*.

Her worst fear was eventually confirmed. A painful compression from a follow-up mammogram ruptured Brenda’s cyst, leading to visible changes on the breast tissue and eventual biopsy. She gave an emotional account of her shock when the mastectomy of her left breast revealed a 10-centimetre tumour that had spread to most of her dissected lymph nodes.

Through her story, Brenda spoke of the value of patient advocacy and the need for more research, better imaging protocols for women with dense breast tissue, and moving away from a “one-size-fits-all” approach to improve diagnosis, treatment, monitoring, and ultimately improved outcomes for patients with lobular breast cancer in the future.

### 10.2. Systemic Therapy

Systemic therapies for ILC were presented in light of the differences between ILC and IDC, including loss of the E-cadherin protein causing a linear growth pattern of cancer cells, higher proportions of estrogen-sensitive and luminal-A subtypes [100], better initial survival but increased recurrence, and more frequent alterations in the *PIK3CA* pathway in ILC [101]. Though ILC is a common cancer, most clinical studies have focused on treatment of the IDC subtype, with only limited data in lobular breast cancer. Evidence guiding the management of systemic therapies for ILC was reviewed.

A 2022 systematic review and meta-analysis showed that ILC is less responsive to chemotherapy in the neoadjuvant setting compared with IDC [102]. However, hormone-sensitive breast cancers are typically treated in the adjuvant setting with genomic tumour profiling widely used to predict risk of recurrence and benefit of chemotherapy. According to a SEER database analysis, 21-gene breast recurrence scores were lower in ILC but still prognostic for breast cancer mortality [103].

In the adjuvant setting, chemotherapy should still be considered standard of care in high-risk patients. A pooled analysis of the phase 3 PlanB and SUCCESS C trials showed that in the subgroups of patients with N2/N3 tumours, anthracycline-containing chemotherapy regimens led to better outcomes in ILC than IDC [104]. ET with AI (letrozole or anastrozole) is preferred over tamoxifen in ILC based on evidence of better DFS with letrozole versus tamoxifen (Breast International Group 1–98 study [105]) and marginally improved OS with anastrozole versus exemestane (MA.27 study [106]). There may be a role for extended ET given that ILC appears to have worse survival outcomes after 10 years. Data are still needed on the magnitude of benefit with CDK4/6 inhibitors in lobular breast cancer.

In the metastatic setting, survival outcomes appear similar in ILC and IDC subgroups treated with CDK4/6 inhibitors as shown in a retrospective analysis [107], providing some evidence to support using CDK4/6 inhibitors as the standard of care is appropriate in this setting. More research focused on ILC is needed, including how distinct molecular signatures in ILC versus IDC will inform targeted therapy options. The phase 2 neoadjuvant LOBSTER study is currently recruiting patients to evaluate the benefit of capivasertib + fulvestrant versus fulvestrant alone in high-risk lobular breast cancer.

### 10.3. Surgical Therapy

Surgical planning for ILC presents unique challenges due to its tendency for diffuse growth, underestimation on imaging, and higher rates of positive margins. The speaker emphasized that when a patient presents with ILC, surgeons anticipate more advanced disease, larger tumours, and higher nodal burden compared with IDC [108]. MRI plays a critical role in preoperative planning, offering greater accuracy in estimating tumour extent than mammography or ultrasound, and is particularly valuable when conventional imaging and clinical findings are discordant [109,110,111]. PET/CT is less sensitive for detecting distant ILC metastases, especially sclerotic bone lesions, making the CT component of particular importance for staging [112].

The principles of surgical management of the breast and axilla in ILC mirror those for IDC with the goal to achieve negative margins while allowing for cosmesis and appropriate management of the axilla based on nodal status. It was noted that women with ILC who undergo lumpectomy or mastectomy have higher rates of positive margins. One study in patients with stage 1–3 ILC who underwent mastectomy showed that the overall margin positivity rate was 10.6% and was 18.7% for tumours larger than 5 cm [113]. However, achieving negative margins reduces the risk of local recurrence in ILC to the risk seen in IDC [110]. There is a potential role of oncoplastic breast surgery to reduce positive margins at initial lumpectomy. Patients with one to two positive nodes with SLNB do not usually need a completion ALND.

In select cases, neoadjuvant chemotherapy may be used to enable surgical downstaging—particularly in patients with pleomorphic lobular carcinoma in situ (LCIS) or HER2+ ILC. Although ILC is generally chemoresistant [110], the speaker presented a case of locally advanced disease where neoadjuvant treatment followed by oncoplastic surgery resulted in no residual invasive or nodal disease, illustrating the importance of individualized planning even within this less responsive subtype [114].

### 10.4. Emerging Research

The Lobular Breast Cancer Alliance is a nonprofit organization bringing together clinicians, researchers, and patient advocates with the common goals to improve research in lobular breast cancer and advance care for patients with ILC. The alliance has identified ways to overcome challenges and advance research in ILC [115]. Recent and emerging evidence in the context of these challenges and opportunities were discussed.

Standardizing diagnosis: There is significant heterogeneity in immunostaining protocols for ILC diagnosis and growth patterns reported in pathology reports [116]. To improve diagnostic accuracy in ILC, the European Lobular Breast Cancer Consortium (ELBCC) has published recommendations on the indications for E-cadherin IHC in cases with inconclusive or uncertain morphology that do not meet the histological criteria for classic ILC [117].

Defining research priorities: Imaging (detection, staging) was identified as a top priority in a recent international survey on ILC [118]. In a prospective, observational cohort study, preoperative breast MRI was shown to significantly reduce (by 6x) reoperations in patients with unilateral needle biopsy-diagnosed ILC [119]. Contrast-enhanced mammogram demonstrated similar performance to MRI [120], offering an option for patients who cannot access MRI. The usefulness of [^18^F]FDG-PET/CT in ILC may be limited due to inconsistent uptake of [^18^F]FDG by ILC tumours, but another approach with 18F-fluoroestradiol (FES) PET/CT may help address the challenges of evaluating ILC with conventional imaging techniques, though this modality is still under study and not routinely used in Canada. Studies on post-mortem tissue may help address challenges with obtaining metastatic tissue samples. In the UPTIDER study, among patients diagnosed with primary ILC (*n* = 6), metastases were found in organs not commonly involved in metastatic breast cancer [121], of which the stomach and visceral fat were notable areas accessible to biopsy. The growth pattern of lobular metastatic lesions showed no clear mass-forming process but complete infiltration by lobular cancer cells [121], underscoring the need to find novel ways to identify lobular carcinoma metastases, such as positron emission mammography. Accelerated partial breast irradiation was noted as an area for further research, as was identifying better gene signatures as predictors for outcomes in lobular breast cancer [122,123]. A detailed multiomic analysis of the tumour microenvironment in ILC suggested immunotherapy or anti-angiogenesis agents could be potential actionable targets in the treatment of ILC [124].

Designing clinical trials focused on lobular breast cancer: Evidence to pique interest in clinical trials for ILC was presented. The phase 2 neoadjuvant ROSALINE study evaluating ET and entrectinib in patients with ILC failed to meet its primary endpoint but the objective response rate (ORR) by MRI was 49% [125]. An exploratory post hoc analysis of the phase 3 PENELOPE-B trial showed a trend in improved survival and invasive disease-free survival with palbociclib at 6 years in the subgroup of patients with lobular breast cancer [126].

Collect data and samples from patients with ILC: There will be opportunities to collect data and samples from Canadian patients with lobular disease through the multicentre, observational cohort LOBCAN study, which is enrolling patients with stage 1 to 3 lobular breast cancer. The study aims to advance understanding of the role of imaging and surgery in ILC, and possibly validate different genomic signatures (e.g., LobSig).

## 11. Survivorship in Breast Cancer—Patient Advocate

The section focused on survivorship issues, such as sexual health, pregnancy, and exercise. The summary appears in Box 6.

Box 6 **Section 11 summary.**

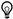
 Section takeaways

- Sexual Health and Breast Cancer—Sexual dysfunction affects the vast majority of survivors (up to 84%), with 11% stopping therapy due to side effects, making sexual health management central to both QoL and treatment adherence.
- Breast Cancer During Pregnancy—With coordinated care, women can safely un-dergo surgery and chemotherapy after the first trimester, achieving outcomes comparable to non-pregnant women while protecting long-term child health.
- Exercise and Breast Cancer—Even modest exercise (90 min/week) improves tolerance to treatment, reduces side effects, and preserves fitness; trials suggest it may also enhance tumour response and reduce recurrence risk.
- Optimal Imaging in Breast Cancer—Mammography alone misses too many cancers in survivors; adding MRI, contrast-enhanced mammography, or ultrasound significantly improves detection, with Canadian data and ACR guidelines supporting combined approaches.

### 11.1. Sexual Health and Breast Cancer

With high rates of survival in early-stage breast cancer, the long-term effects of treatment on QoL are increasingly important. One of the most challenging yet often overlooked issues is sexual health. OFS, AI, tamoxifen, and chemotherapy-induced menopause can all profoundly affect sexual function, leading to dryness, pain, reduced arousal, and loss of desire [127,128]. A Canadian survivorship study showed that 84% of women reported significant sexual dysfunction and 11% admitted to stopping their medication because of side effects [129]. This underscores how sexual health is not simply a quality-of-life issue but one directly tied to adherence and long-term cancer outcomes. Patients may tolerate one treatment better than another, making education, shared decision-making, and side-effect mitigation critical for keeping women on life-prolonging therapy.

Management of sexual dysfunction requires a multimodal approach [130]. While non-hormonal strategies such as pelvic floor physiotherapy, dilators, lubricants, and moisturizers provide benefit, data increasingly support the safety of local vaginal estrogen in most breast cancer survivors, with no demonstrated increase in recurrence or mortality in large systematic reviews [131]. Newer therapies are expanding choices: prasterone (vaginal dehydroepiandrosterone) improves dryness and pain, and in women on AI its conversion to estrogen is largely blocked, making it a practical option [132]. Ospemifene, an oral selective estrogen receptor modulator (SERM), is especially exciting because it strengthens vaginal tissue while blocking estrogen’s effects in the breast. In Europe it is already approved for women with a history of breast cancer once treatment is finished, though in Canada its label still lists hormone-dependent cancers as a contraindication [133,134]. Alongside these pharmacologic strategies, sexual counselling and therapy remain essential—helping women and their partners adjust expectations and regain intimacy. The overarching message is clear: supporting sexual health is central to survivorship, adherence, and overall wellbeing, not an optional add-on to cancer care.

### 11.2. Breast Cancer During Pregnancy

Breast cancer diagnosed during pregnancy is rare but increasingly seen as women delay childbearing. It is important to distinguish this from postpartum breast cancer, which carries a different biology and often a poorer prognosis [135]. When cancer arises during pregnancy, outcomes are generally comparable to non-pregnant women provided treatment follows standard protocols. Diagnosis can be made safely with ultrasound, and mammography may be added without risk to the fetus. Surgery, including SLNB, is feasible at any stage, while radiation is usually postponed until after delivery.

When it comes to systemic therapy, chemotherapy is the cornerstone for many of these women, who are often young and present with aggressive subtypes. It is strictly avoided in the first trimester, when fetal organs are forming and the risk of malformation is highest [136]. From the second trimester onward, however, regimens such as anthracycline-based therapy and taxanes can be given safely, with malformation rates no higher than the general population [137]. Treatment does carry added risks of low birth weight, early delivery, and maternal complications such as hypertension, so pregnancies are managed closely in high-risk clinics. Physiologic changes in pregnancy alter how drugs are metabolized, raising concerns that women might be under-dosed, but large registry studies comparing hundreds of pregnant and non-pregnant patients have shown no compromise in survival when full, weight-based doses are used. To balance safety for mother and baby, chemotherapy is usually stopped by 35 weeks to allow time before delivery [136].

Beyond the technical aspects of care, these women face profound personal challenges—navigating cancer treatment while expecting a child. Concerns about fertility, breastfeeding, body image, and depression are common, and many mothers worry about the long-term health of their children. The key message is one of hope: with coordinated multidisciplinary care, women can be treated effectively during pregnancy without compromising their child’s long-term health, and they deserve not only oncologic excellence but also strong emotional and social support for this uniquely difficult journey.

### 11.3. Exercise and Breast Cancer

Exercise has well-established benefits for the general population, and these extend meaningfully to people living with and beyond breast cancer. For cancer survivors, even 90 min of moderate activity per week can make a meaningful difference [138]. Beyond the usual gains in mood, energy, and fitness, exercise brings cancer-specific benefits: women who stay active during treatment are more likely to complete chemotherapy at the planned dose and less likely to stop early. It can ease fatigue, nausea, and joint pain from aromatase inhibitors, and importantly, it helps patients maintain their cardiorespiratory fitness through treatment—holding steady at a time when fitness would otherwise decline.

The research is catching up to what many patients already feel in practice. A landmark trial in colon cancer showed that three years of structured aerobic exercise after chemotherapy improved DFS, cutting recurrence risk by 30% (HR 0.70) [139]. While breast cancer trials have been smaller, they offer promising signals: in the BENEFIT German neoadjuvant study, aerobic exercise improved tumour response in hormone-positive women, and both aerobic and resistance training reduced the chance of stopping chemotherapy early [140]. What exercise has not yet been shown to do is prevent anthracycline-related heart damage in humans, despite encouraging animal data. The practical message remains simple and hopeful: encourage women to avoid inactivity, adjust activity to “good” and “bad” days, and aim for a mix of walking, resistance training, and flexibility work. Even if fitness doesn’t improve during chemotherapy, preserving strength, resilience, and QoL is a powerful achievement.

### 11.4. Optimal Imaging in Breast Cancer

Early detection of new or recurrent breast cancers is central to improving survival. Annual mammography remains the worldwide standard and is effective at finding most second cancers at stage 0 or I, with meta-analyses showing a 17–28% reduction in mortality when recurrences are identified through screening rather than at clinical exam [141]. Still, mammography performs less well in survivors. Sensitivity is only ~65% compared to 77% in average-risk women, and interval cancers—those diagnosed between scheduled screens—occur more than twice as often [142]. This reduced accuracy reflects both the scarring and architectural distortion left by prior surgery or radiation, which can mask small lesions, and the added difficulty of detecting tumours in dense breast tissue [143].

Supplemental imaging helps close this gap. MRI is the most sensitive option and can identify cancers hidden on mammography with detection rates up to 85–100% in trials [144,145]. Canadian experience reflects this. At the Ottawa Hospital and UBC, women with a personal history of breast cancer and a normal mammogram had 20 cancers per 1000 detected with MRI and 13.6 per 1000 with ultrasound, far above what mammography alone could achieve. Contrast-enhanced mammography is emerging as a lower-cost alternative, with detection rates of 9–14 per 1000 and high patient acceptability, while ultrasound—more widely available—adds another 4–6 cancers per 1000 after a normal mammogram [146,147,148,149]. The American College of Radiology now recommends annual surveillance with both mammography and MRI for women with a personal history of breast cancer, and these policies are mirrored in Ottawa where MRI is offered every two years under age 50 and ultrasound is substituted beyond that age when MRI is less accessible. Taken together, the message is clear. For many survivors, mammography alone is not enough. Integrating MRI, contrast-enhanced mammography, or ultrasound into surveillance can improve early detection, reduce the burden of advanced-stage recurrences, and give women the best chance at long-term survival.

## 12. Best Poster Abstract Submission 2025

The best posters included topics on surgical techniques, screening, and recurrence rates. A summary appears in Box 7.

Box 7 **Section 12 summary.**

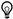
 Section takeaways

- Upfront TAS—TAS is increasingly replacing full axillary dissection in node-positive HR+/HER2− disease, sparing 77% of women from ALND while maintaining low short-term recurrence.
- Cost-Effectiveness of Breast Cancer Screening—Lifetime modelling shows start-ing mammography at 40 is not only life-saving but cost-saving, with annual screen-ing providing excellent value at <$25,000 per death averted.
- DCIS Margins and Re-excision—Women with margins of 1–1.9 mm who receive radiation have recurrence rates similar to those with ≥2 mm, suggesting re-excision can often be avoided, while margins <1 mm still warrant surgery.

### 12.1. Upfront Tailored Axillary Surgery for Clinically Node-Positive HR+/HER2− Breast Cancer: A Population-Based Cohort

ALND has been the standard for clinically node-positive breast cancer treated with upfront surgery, yet its long-term morbidity and tendency to remove more nodes than necessary have prompted interest in alternatives. Tailored axillary surgery (TAS) targets palpably or radiologically abnormal nodes along with sentinel nodes, always combined with adjuvant nodal radiation. Early retrospective series have suggested oncologic safety, and the NCCN now supports selective use [150]. This Alberta population-based study examined real-world uptake in HR+/HER2− patients with clinical N1 disease between 2017 and 2024. Of 777 eligible patients, 124 (16%) underwent TAS, with use rising to 40% by 2024.

TAS retrieved a median of four nodes, and while 23% required conversion to ALND, 77% were spared a full dissection. At a median follow-up under two years, only one axillary recurrence was observed (1%), suggesting short-term oncologic safety. Rehabilitation referrals were made for 25% of TAS patients compared with 46% after ALND, most often for range of motion restriction or lymphedema. Overall, TAS is increasingly used, allows most patients to avoid ALND, and shows low recurrence rates, though morbidity may be greater than assumed. A forthcoming prospective cohort, the ARMOR study, will further clarify its impact on QoL.

### 12.2. Cost-Effectiveness of Breast Cancer Screening Using Digital Mammography in Canada

While breast cancer screening has long been recognized for its ability to detect disease earlier and improve outcomes, its economic impact in the context of rising treatment costs remains less well defined. This study, published in *JAMA Open*, used the Canadian OncoSim breast micro-simulation model to evaluate the cost-effectiveness of population-based screening strategies with digital mammography [151]. A modelled birth cohort of 1.23 million women born in 1975 was followed over their lifetime to compare scenarios including no screening, biennial screening from ages 50–74 (the current standard), earlier initiation at age 40, hybrid schedules, and annual screening. Importantly, unlike prior analyses restricted to shorter horizons, this model incorporated lifetime outcomes and detailed Canadian cost inputs spanning screening, diagnosis, systemic therapy, radiation, and health system expenditures.

The results demonstrated that earlier and more frequent screening not only improved clinical outcomes but also reduced overall system costs. Initiating screening at age 40 led to more cancers detected at earlier stages and prevented additional deaths compared with starting at 50. Although upfront imaging costs increased, treatment costs fell substantially, making all strategies cost-saving compared with no screening. When the economic trade-off between added costs and added benefits was calculated, annual screening from 40–74 was especially favourable—amounting to only $25,000 for each additional death averted and $1500–$4000 for each additional quality-adjusted life year gained, well below the thresholds typically considered good value in healthcare ($50,000–$100,000). Several strategies even saved money outright while improving outcomes. In summary, digital mammography screening in Canada is not only effective but economically advantageous, simultaneously reducing expenditures and saving lives.

### 12.3. 10-Year Local Recurrence Rates Following Selective Omission of Re-Excision for Patients with DCIS and Margins <2 mm

The question of how wide a surgical margin should be for DCIS has been a source of debate for years. Current guidelines recommend a 2 mm margin to optimize local control after breast-conserving surgery, but the evidence supporting this cut-off is limited, especially for patients whose margins fall just short of the threshold [152,153]. As a result, many women are sent back to the operating room for a re-excision, even when their margins are only slightly under 2 mm, leading to additional procedures, stress, and recovery time. This Alberta study set out to ask whether re-excision is truly necessary in all such cases, particularly when radiation therapy is already part of the treatment plan.

Looking at nearly 470 women treated between 2010 and 2014, with follow-up extending beyond 10 years, the study found that recurrence rates were reassuringly low—around 6% at 10 years for patients with margins ≥2 mm and 9% for those with margins 1–1.9 mm. Crucially, in patients with close margins who received radiation, outcomes looked very similar to those with wider margins. In contrast, women with margins <1 mm had much higher recurrence, with rates approaching 20%, regardless of radiation. The message is clear: for patients with margins just shy of the 2 mm standard, another surgery may not always be necessary if radiation is planned, sparing many women from additional operations without compromising long-term outcomes. However, for margins under one millimetre, the risk remains too high to forgo re-excision.

## 13. Central Nervous System Metastases

The section focused on brain metastases including radiotherapy, systemic therapy, and screening. A summary appears in Box 8.

Box 8 **Section 13 summary.**

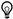
 Section takeaways

- Post-operative radiation therapy with cavity SRS is recommended in all resected metastases. SRS alone followed with serial MRI is appropriate in non-surgical cases with good performance status and <10 lesions and WBRT should be considered in patients with >10 brain metastases, leptomeningeal disease, or pachymeningeal disease.
- Higher quality data are needed to guide clinical practice and determine whether screening for brain metastases can help patients with metastatic breast cancer live longer with improved QoL.
- Systemic therapies can be highly effective in treating both stable and active brain metastases for patients with HER2+ metastatic breast cancer. While there have been advances in clinical trial design to include patients with brain metastases, the inclusion criteria for certain breast cancer subtypes (e.g., TNBC) are still quite restrictive.
- Clinical trials to inform optimal sequencing of systemic and local therapies to treat patients with metastatic breast cancer and brain metastases are required.

### 13.1. Radiotherapy for the Management of Breast Cancer Brain Metastases

Brain metastases are a major cause of morbidity and mortality and occur in a significant proportion of patients with metastatic breast cancer, with similar incidences reported in HER2+ (31%) and TNBC (32%) compared with HR+/HER2− (15%) disease [154]. The role of stereotactic radiosurgery (SRS) and whole brain radiation therapy (WBRT) in the context of surgical and non-surgical approaches to treat brain metastases was discussed.

Surgery should be considered for solitary/large symptomatic brain metastasis in patients with good performance status [Eastern Cooperative Oncology Group (ECOG) 0–2] and reasonable prognosis. Post-operative cavity SRS should be considered in all resected metastases for better QoL [155].

In non-surgical cases, SRS provides long-term control with minimal collateral damage (i.e., good sparing of normal brain and other nearby structures). SRS should be considered first as it is associated with reduced risk of neurocognitive decline with similar OS compared with WBRT [156]. SRS alone with serial MRI follow-up is most appropriate for patients with good performance status and prognosis, stable/controlled extracranial disease, and fewer than 10 lesions. SRS is well tolerated, but there is a risk of late grade 3+ radiation necrosis in ~5–10% of patients [157,158]. The International Stereotactic Radiosurgery Society Practice Guideline does not specifically recommend adding WBRT to SRS given this approach did not improve OS and resulted in worse QoL and neurocognitive outcomes [159].

WBRT provides temporary control but can damage normal brain tissue and lead to neurocognitive decline in 30–50% of patients. WBRT should be considered in patients with diffuse discrete brain metastases (≥10), leptomeningeal metastatic disease (LMD), or pachymeningeal disease. Patients suitable only for WBRT can have additional SRS to lesions where local control is important. This approach is supported by findings from a meta-analysis showing that adding SRS to WBRT increases local control but does not improve survival [160].

Systemic therapy shows promise for the treatment of CNS metastases. Recently the HER2-targeted ADC, T-DXd, demonstrated efficacy in patients with brain metastases [16,161,162], while the phase 2 DEBBRAH trial and the ROSET-BM multi-centre retrospective cohort study showed promising efficacy of T-DXd in patients with LMD, which historically has dismal prognosis [161]. Best support care (BSC) is considered for patients with very poor performance status (ECOG 3–4) and very limited prognosis, especially if they have already received previous WBRT.

Future directions included investigating the integration of RT and T-DXd in patients with HER2+ or HER2-low breast cancer and CNS metastases, adaptive boost after SRS for large (>3 cm) metastases to maximize local control without increasing risk of radiation necrosis, and safe treatment for local progression after SRS with staged adaptive re-radiation with SRS.

### 13.2. Screening (Why or Why Not?)

Evidence for and against screening for brain metastases in HER2+ disease were discussed. Reasons in support of screening included the high incidence of brain metastases and poor prognosis in HER2+ metastatic breast cancer, the availability of effective treatments, and the potential to improve patient outcomes. A third of patients with HER2+ metastatic breast cancer will develop brain metastases during their lifetime [154]. However, the incidence in clinical practice may be even higher when accounting for screening for asymptomatic brain metastases. The presence of brain metastases is associated with shorter survival and adverse QoL [163]. If brain metastases are found with screening, systemic therapies are effective in treating patients as shown in the DESTINY-Breast12 (T-DXd) and HER2CLIMB (tucatinib + T-DXd + capecitabine) studies [16,164]. Data also suggest that implementing effective therapies in patients with brain metastases can prevent the development of brain metastases in the future; this was demonstrated in an exploratory analysis of the HER2CLIMB trial [164]. In clinical practice clinicians are seeing their patients responding well to systemic treatment, bringing into question whether screening for brain metastases at earlier stages would translate into improved survival for patients. One study by Cagney et al. 2018 showed that breast cancer (not typically screened for CNS metastases) versus non-small cell lung cancer (typically screened for CNS metastases) presented with more advanced intracranial disease with larger, more numerous, and symptomatic brain metastases [165]. From the patient’s perspective, screening would allow them to better understand what is going on in their bodies, something patients generally want to know [166].

Reasons against screening for CNS metastases were the lack of high-quality evidence, potential risks of screening, high costs, and mixed guidelines as recently reviewed by Jerzak et al. [167]. Miller et al. 2003 showed no difference in survival among patients with metastatic breast cancer with occult versus symptomatic brain metastases [168]. While outdated these data were influential in raising questions about the benefit of CNS screening. Potential risks associated with brain metastases screening included anxiety, detriment to QoL, and the potential risks of unnecessary treatment and associated toxicities with WBRT (cognitive impairment), SRS (radiation necrosis), and surgery. Furthermore, uncovering asymptomatic brain metastases may limit treatment options as many breast cancer trials still exclude patients with CNS metastases [169]. With the high costs of serial MRI screening, any limited resources available should be allocated to interventions with proven benefits. To date there is no consistent recommendation on screening from international guidelines and clinical studies are underway to evaluate MRI screening for brain metastases in metastatic breast cancer [167].

### 13.3. Brain Metastases Systemic Therapy Overview

Clinical evidence of systemic therapies for the treatment of brain metastases was reviewed. Most data were in the HER2+ subtype. The HER2CLIMB study was one of the first trials to include patients with metastatic HER2+ breast cancer and active brain metastases and showed that third-line treatment with tucatinib + capecitabine + trastuzumab was associated with a significant PFS benefit and prolonged new brain lesion-free survival [170], a relevant endpoint for patients.

Data for T-DXd in patients with HER2+ breast cancer and brain metastases has proven that large molecule systemic therapies can penetrate the blood–brain barrier and are highly effective in treating both stable and active brain metastases. In the second-line setting where T-DXd is currently the standard of care for HER2+ breast cancer, DESTINY-Breast03 showed that median PFS was 14.1 months with T-DXd versus 2.8 months with T-DM1 in patients with brain metastases [171]. Screening for brain metastases on a subtype-specific basis at the time of extracranial disease progression may be appropriate based on these data. Interestingly, HER2+ expression in primary and metastatic tumours can vary (e.g., brain metastases were found to be HER2+ even if the primary tumour was not) [172], suggesting the need to better understand brain metastases to develop effective therapies.

There is currently very limited evidence for use of systemic therapy among patients with HER2-low metastatic breast cancer and brain metastases. In the DESTINY-Breast04 trial, only ~5% of enrolled patients had previously treated/stable brain metastases at baseline [34]. There is an unmet need to develop novel systemic therapies with CNS activity for patients with TNBC. While more clinical trials are allowing enrollment of patients with brain metastases, many require patients to have stable brain metastases for 28 days prior to enrollment, which is not a luxury of time that most patients with TNBC have. In the ASCENT study, a subgroup analysis of patients with stable/previously treated brain metastases showed that the median PFS was quite short (2.8 months) with SG [173].

Newer, “thought-provoking” data for non-approved therapies was also presented. The results of the DEBBRAH trial of T-DXd in patients with LMD were described as “unprecedented” given the dismal prognosis of these patients seen in clinical practice, though treatment outside of the DESTINY-Breast03 or DESTINY-Breast04 indication has not yet been approved in Canada [161]. Patritumab deruxtecan (HER3-DXd) is a new HER3-targeted ADC [174]. TUXEDO-3 demonstrated that HER3-DXd was effective in patients with heavily pre-treated metastatic breast cancer and active brain metastases, irrespective of breast cancer subtype (HER2+, luminal, triple-negative) [175], findings that were considered exciting in the context of the high unmet need in patients with brain metastases.

## 14. Novel Advances in Breast Cancer

The section focused on new agents for the treatment of breast cancer as well as imaging techniques for diagnosis. A summary appears in Box 9.

Box 9 **Section 14 summary.**

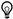
 Section takeaways

- New ADC are expanding targeted therapy options in breast cancer, showing activity across different tumour types and raising new questions about sequencing and toxicity.
- Recent advances in immunotherapy, including novel checkpoints, bispecific antibodies, and cell-based strategies, are offering new treatment avenues for breast cancer.
- Emerging technologies like tomosynthesis, contrast-enhanced mammography, and artificial intelligence-driven detection are improving breast cancer imaging by increasing diagnostic accuracy and addressing previous screening limitations.

### 14.1. Antibody-Drug Conjugates

ADCs combine a target-specific antibody, a linker, and a cytotoxic payload. Most modern agents use a cleavable linker to carry a single payload, though dual-payload designs are emerging. T-DM1, approved in 2013 for HER2+ breast cancer, was the first ADC introduced in this setting. Since then, subsequently approved agents have included T-DXd, targeting HER2 and incorporating a topoisomerase I inhibitor payload, and SG, targeting TROP2 also with a topoisomerase I inhibitor payload.

Multiple new ADCs are currently in development. Disitamab vedotin (RC48) targets HER2 with a microtubule-disrupting payload and is being studied in HER2+ and HER2-low breast cancer, as well as other HER2-expressing solid tumours. Trastuzumab duocarmazine (SYD985) and ARX788 are additional HER2-directed agents in early development.

TROP2-targeted agents include Dato-DXd, featuring a unique toxicity profile with higher rates of stomatitis, and sacituzumab tirumotecan (sac-TMT), which has been shown to improve PFS versus chemotherapy in locally recurrent or metastatic TNBC [176]. Both agents employ a topoisomerase I inhibitor payload and exert their activity in part through an antitumour bystander effect.

HER3 is expressed in up to 30–50% of breast cancers and is associated with poor outcomes. HER3-DXd, a HER3-targeted ADC, has shown pathologic complete response and ORR comparable to multi-agent chemotherapy in early-stage studies [177]. Other investigational targets include B7-H4, LIV1, Nectin-4, and CEACAM5, with agents such as puxitatug samrotecan (P-Sam) and tusamitamab ravtansine under evaluation.

Key challenges include optimal sequencing after ADC failure, duration of therapy, management of distinct toxicity profiles, biomarker refinement for patient selection, and equitable access. Ongoing trials are expanding ADC use across subtypes and into earlier disease stages, emphasizing their growing role in reshaping the treatment landscape for breast cancer.

### 14.2. Immunotherapy and Cell Therapy

Standard immunotherapy in breast cancer currently includes pembrolizumab combined with neoadjuvant chemotherapy for node-positive, early-stage triple-negative disease and for PD-L1+ metastatic disease with a combined positive score (CPS) greater than 10.

For TNBC, one promising avenue that has recently developed is vascular endothelial growth factor (VEGF) inhibition combined with immunotherapy. Early trials of bevacizumab, a VEGF inhibitor, in breast cancer improved response rates but not survival. However, recent studies combining anti-VEGF agents with programmed cell death protein 1 (PD-1) inhibition have shown high activity, with one trial showing an ORR of 63% [178]. Bispecific antibodies targeting both VEGF and PD-1 or PD-L1, such as ivonescimab and BNT327, have demonstrated ORR exceeding 70% in first-line metastatic TNBC, with tolerable toxicity profiles [179,180]. Another approach for TNBC includes SGN-PDL1V, a PD-L1–targeted ADC showing early evidence of activity [181].

For HER2+ disease, one novel approach is a combination of a HER2-targeted bispecific antibody, zanidatamab, with a CD47 inhibitor, evorpacept, to enhance macrophage-mediated phagocytosis. This treatment showed promising activity in HER2+ disease, with an ORR over 50%, though a less pronounced effect in HER2-low tumours [182].

Additional investigational targets include TIGIT and LAG-3 immune checkpoints, anti-CCR8 antibodies to deplete regulatory T cells, and personalized cancer vaccines. Cell-based therapies, while established in hematologic malignancies, face challenges in solid tumours. A first-in-human study of HER2-targeted chimeric antigen receptor (CAR) macrophages achieved proof-of-concept disease control in heavily pretreated HER2-positive breast cancer, with a best overall response rate of 44% but short durability [183].

Current investigation focuses on defining optimal drug combinations, refining predictive biomarkers, and determining the sequencing of novel immunotherapies, all while managing toxicity.

### 14.3. Novel Imaging Techniques in Breast Cancer Diagnosis

Mammography remains the cornerstone of breast cancer screening, with historical evidence from large, randomized trials showing a 24% reduction in mortality and observational data confirming lower rates of advanced disease [184]. Regular participation in screening provides the greatest survival benefit, and Canadian data demonstrate its cost-effectiveness, particularly with annual screening from age 40 to 74 [151]. Despite these benefits, limitations include reduced sensitivity in dense breasts, false positives leading to unnecessary biopsies, and detection of indolent lesions.

To address these longstanding challenges, recent advances in imaging technology have aimed to fill critical gaps. The introduction of digital breast tomosynthesis (DBT) has markedly improved cancer detection rates, particularly in women with dense breasts, by generating 3D reconstructions that reduce tissue overlap. Meta-analyses suggest that DBT lowers interval cancer rates, with definitive trial results pending [185].

Contrast-enhanced mammography (CEM) is an imaging technique that combines a standard mammogram with an intravenous injection of iodine-based contrast dye, allowing detection of abnormal neovascularity characteristic of breast cancer. Meta-analyses show comparable diagnostic accuracy to MRI for tumour extent [186], making CEM a viable alternative when MRI is unavailable or contraindicated. CEM may improve workflow efficiency in symptomatic assessment and, in dense breasts, matches abbreviated MRI for cancer detection rates, with low rates of severe contrast reactions [187].

Artificial intelligence-based, computer-aided detection systems now automatically extract complex imaging features, assisting in lesion detection, characterization, triage, and workload reduction. A randomized trial demonstrated that artificial intelligence use increased cancer detection by 29% and was safe for integration into screening programmes [188]. Future priorities include assessing the impact on mortality, further refining cost-effectiveness, and optimizing clinical integration of these technologies.

## 15. Advocacy in Breast Cancer

The section focused on patient advocacy with a summary appearing in Box 10.

Box 10 **Section 15 summary.**

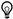
 Section takeaways

- When experts, advocates, and communities unite, breast cancer care moves beyond treatment towards equity, empowerment, and real change.

### 15.1. Breast Cancer Canada/REAL Alliance

This presentation showcased the unique partnership between the REAL Alliance and Breast Cancer Canada—two groups working hand-in-hand to change the landscape of breast cancer in Canada. REAL Alliance is a network of leading oncologists from across the country, supported by Breast Cancer Canada, which has quickly become a national voice. In two years, they have published guidance documents on staging and HER2+ disease, giving clinicians trusted, Canadian-specific recommendations. REAL Alliance is also involved in health technology assessment (HTA) submissions, ensuring clinicians’ voices are heard when new therapies are reviewed for approval and funding. This collective work is critical for moving evidence-based treatments from research into clinical care. With a new membership programme launching soon, even more clinicians will be able to contribute to guidance documents, education, and HTA feedback. National guidance publications on hormone receptor–positive (early and late), triple-negative, and HER2+ update are anticipated this year.

Breast Cancer Canada complements this expert work with a strong patient focus. Their tools like Progress Connect give newly diagnosed patients clear roadmaps for their care, while the updated version even interprets pathology reports to make them less overwhelming. The Progress Tracker study collects real-world patient experiences over decades, creating a rich source of insight for research. Breast Cancer Canada also partners with leaders like Dr. Nancy Nixon on new resources such as a metastatic breast cancer app, giving patients reliable information and a supportive community. Beyond research and tools, Breast Cancer Canada also funds dozens of projects nationwide and runs powerful awareness campaigns like their award-winning *Breast Cancer Won’t Wait*, which has toured major Canadian cities.

REAL Alliance and Breast Cancer Canada combine expert knowledge and the patient voice to ensure clinical research is applied to patient care to improve patient outcomes.

### 15.2. Rethink Breast Cancer

Rethink Breast Cancer brings system change through community-building. Founded in 2001 to engage younger women in breast health and research, Rethink discovered that young women with breast cancer were reaching out, often feeling isolated, misunderstood, and unsupported. In response, Rethink built communities through retreats, conferences, films, and creative campaigns, while developing age-appropriate resources co-created with patients and experts. Their Give a Care programme, for instance, provides practical, thoughtful care packages that help newly diagnosed patients feel seen. Over time, Rethink evolved from raising awareness to tackling policy and access barriers. Since 2012, they have submitted several HTAs and led advocacy campaigns that improved access to treatments and shaped national policy. Their landmark *Metastatic Breast Cancer in the Dark* campaign became a blueprint for their model: start with building community, listen to patient needs, gather evidence, consult with all stakeholders, and then amplify patient voices through bold, strategic advocacy. This approach has led to greater transparency and benchmarks for drug negotiations.

Rethink continues to focus on three priority groups: young people with breast cancer, those living with metastatic disease, and individuals facing systemic barriers to care. With a small but nimble team and a network of patient collaborators, they are known as “friendly disruptors”—combining compassion with strategy to create bold, evidence-based advocacy. Their message to clinicians was clear: connect patients to Rethink’s resources, collaborate openly, and work together toward the shared goal of improving outcomes and experiences for those facing breast cancer.

### 15.3. Patient Groups: Your Partners on the Ground

This panel brought together voices from across Canada’s breast cancer advocacy community—Rethink Breast Cancer, the Canadian Breast Cancer Network (CBCN), and the Olive Branch of Hope—along with patient advocates who shared personal experiences. The conversation underscored both the challenges patients face and the power of community-driven support.

Aya McMillan, a young survivor and Rethink collaborator, spoke candidly about the shock and isolation of being diagnosed in her 30s: sitting in waiting rooms surrounded by seniors, scrambling to make life-altering decisions quickly, and feeling alone until she found connection through Rethink’s community. Jen Gordon expanded on how Rethink now provides digital-first support for younger patients—from large online communities to therapist-led virtual support groups and peer programmes that are accessible nationwide without waitlists.

Leila Springer shared her journey as a Black woman diagnosed in 1999 and how feeling unseen led her to establish the Olive Branch of Hope. She described the importance of culturally specific spaces where women can share experiences without judgement, and urged clinicians to “see me, hear me, feel me”—recognizing the fear and mistrust many women of colour carry into clinical settings. Her organization provides education, advocacy, and empowerment, ensuring women are not only supported but better equipped to face their journey.

Rebecca Armstrong highlighted CBCN’s role in empowering patients through education and navigation, helping them understand diagnoses, treatment options, and how to self-advocate with their care teams. She emphasized that knowledge restores a sense of control, allowing patients to participate actively in decisions—from everyday treatment discussions to complex topics like oligometastatic disease.

Together, the panel reinforced that medical care alone is not enough. Patients need to be understood as whole people, connected to peers, supported with culturally relevant resources, and armed with the knowledge to advocate for themselves. For clinicians, the message was clear: refer patients to these organizations, because education, empathy, and community can transform a frightening diagnosis into a more supported and empowered journey.

## 16. Concluding Remarks

CBCS 2025 provided a comprehensive overview of contemporary advances in breast cancer care, grounded by the perspectives of patients whose experiences frame our clinical purpose. Sessions spanning systemic therapy, surgery, radiation oncology, imaging, genetics, global health, CNS metastases, survivorship, and emerging technologies and mechanisms of action have highlighted the rapidly evolving evidence that informs our practice. Challenging case discussions and new Canadian research presented through abstract sessions further underscored the importance of multidisciplinary collaboration. As we conclude, we reaffirm our commitment to integrating scientific innovation with patient-centred priorities to advance outcomes across the breast cancer continuum.

## Figures and Tables

**Table 1:** Top 3 papers in breast cancer 2025.

**Top Papers in Radiation Therapy**	
1. EUROPA [1]	RT (~85% PBI) offers better HRQOL GHS preservation than ET as well as lower incidence of treatment-related AEs at 24 months
2. HypoG-01 [2]	Moderately hypofractionated loco-regional RT showed mild early AEs with no safety concerns, supporting its use in women receiving nodal irradiation.
3. TROG 20.03 AVATAR [3]	SABR should be considered for patients with oligoprogressive ER+/HER2– breast cancer as a strategy to safely delay changing systemic therapy, allowing continued benefit from CDK4/6 inhibitor plus AI treatment
**Top Papers in Surgical Oncology**	
1. *More is More:* BRCA BCY consortium study [4]	RRM in g*BRCA1/2* carriers with stage I-III invasive breast cancer is associated with a significant survival advantage
2. *Less is More:* INSEMA [5]	Omission of SLNB in cT1N0 HR+/HER2- breast cancer is associated with reduced arm morbidity
3. *Less is the same:* COMET [6]	Women with low-risk DCIS participating in COMET and randomized to active monitoring did not have higher rates of invasive cancer at 2 years compared to those randomized to GCC
**Top Papers in Systemic Therapy**	
1. Mutational mayhem in ER + HER2-low metastatic breast cancer [7,8,9,10,11,12]	Our challenge is standardized biomarker testing and balancing efficacy with real-world tolerability
2. Treatment of TNBC and HER2+ [13,14,15]	Growing trend toward dual- and triple-agent strategies will require thoughtful consideration of toxicity, treatment history, and individual patient factors
3. Treatment of Brain Metastases [16]	Choosing therapies based on intracranial vs. systemic progression remains nuanced, and real-world sequencing continues to be a clinical challenge

Abbreviations: AEs = adverse events; AI = aromatase inhibitors; CDK4/6 = cyclin-dependent kinase 4 and 6; DCIS = ductal carcinoma in situ; ER = estrogen receptor; GCC = guideline-concordant care; ET = endocrine therapy; g*BRCA1/2* = germline breast cancer gene 1 or 2; GHS = global health score; HER2 = human epidermal growth factor receptor 2; HR = hormone receptor; HRQOL = health-related quality of life; PBI = partial breast irradiation; RRM = risk reducing mastectomy; RT = radiotherapy; SLNB = sentinel lymph node biopsy; SABR = stereotactic ablative body radiotherapy; TNBC = triple-negative breast cancer.

**Table 2:** Debate summary.

Should ctDNA Be Used in Early Breast Cancer?	
YES	NO
✔ Identifies hidden relapse risk early.	✘ No proven benefit from acting on test results.
✔ Highly sensitive with newer tests.	✘ Hard to detect ctDNA reliably in early-stage disease.
✔ Could reduce unnecessary treatment for some patients.	✘ No treatments proven to work based on ctDNA in early disease.
✔ Offers peace of mind to patients after treatment.	✘ May cause anxiety without clear action plans.

## Data Availability

No new data were created or analyzed in this study. Data sharing is not applicable to this article.

## Supplementary Materials

The following supporting information can be downloaded at: https://www.mdpi.com/article/10.3390/curroncol33010015/s1, Table S1: Canadian Breast Cancer Symposium 2025 Agenda.

## Abbreviations

The following abbreviations are used in this manuscript:

ALND	axillary lymph node dissection	LYMPHA	lymphatic microsurgical preventing healing approach
ARM	Axillary Reverse Mapping	MRD	minimal residual disease
ASCO	American Society of Clinical Oncology	MRI	magnetic resonance imaging
BCS	breast-conserving surgery	NCCN	National Comprehensive Cancer Network
BI-RADS	breast imaging reporting and data system 2	NSCLC	non-small cell lung cancer
BMI	body mass index	OFS	ovarian function suppression
BRCA1/2	breast cancer gene 1 or 2	ORR	overall response rate
BSC	best support care	OS	overall survival
CBCS	Canadian Breast Cancer Symposium	PD-1	programmed cell death protein 1
CDK4/6i	cyclin-dependent kinase 4/6 inhibitor	PD-L1	programmed cell death ligand 1
CEM	contrast enhanced mammography	PET	positron emission tomography
CNS	central nervous system	PFS	progression-free survival rate
CPS	combined positive score	Pi3K	phosphatidylinositol 3-kinase
CRRM	contralateral risk-reducing mastectomy	PR	progesterone receptor
CT	computed tomography	QoL	quality of life
ctDNA	circulating tumour DNA	RRM	risk-reducing mastectomy
DBT	digital breast tomosynthesis	RRSO	risk-reducing salpingo-oophorectomy
DCIS	ductal carcinoma in situ	RT	radiotherapy
dato-DXd	datopotamab deruxtecan	SABR	stereotactic ablative body radiotherapy
DFS	disease-free survival	SEER	surveillance, epidemiology, and end results
ECOG	Eastern Cooperative Oncology Group	SERD	selective estrogen receptor degrader
ER+/-	estrogen receptor positive/negative	SG	sacituzumab govitecan
ESMO	European Society of Medical Oncology	SLNB	sentinel lymph node biopsy
ESR1	estrogen receptor 1	SRS	stereotactic radiosurgery
ET	Endocrine therapy	TAS	tailored axial surgery
gBRCA	germline breast cancer gene	T-DM1	trastuzumab emtansine
HER2+/–	human epidermal growth factor receptor-2-negative/positive	T-DXd	trastuzumab deruxtecan
HF	hypofractionated	THP	taxane-trastuzumab-pertuzumab
HR	hazard ratio	TNBC	triple-negative breast cancer
HR+/-	hormone receptor-positive/negative	TROP2	trophoblast cell surface antigen 2
HRQOL	health-related quality of life	VEGF	vascular endothelial growth factor
HTA	health technology assessment	WBRT	whole-brain radiotherapy
IDC	invasive ductal carcinoma		
IHC	immunohistochemistry		
ILC	invasive lobular carcinoma		
ILR	immediate lymphatic reconstruction		
JAMA	Journal of the American Medical Association		
