# Supplementary material for: The Canadian Breast Cancer Symposium 2025: Meeting Report
Source: Curr Oncol. 2025 Dec 27;33(1):15. doi: 10.3390/curroncol33010015 (PMC12839805; doi:10.3390/curroncol33010015)
Supplement: Supplementary file 1 [file curroncol-33-00015-s001.zip › curroncol-4053149-supplementary.pdf]

Friday, June 20

07:00 Poster Viewing, Breakfast, Registration and Audience Best Abstract Voting

07:45 Non-Accredited Symposium

09:00 Morning Remarks
Christine Brezden-Masley & Tulin Cil

Session VIII: Optimization Strategies for Lobular Breast Cancer

Moderator(s): ***Ana Lohmann***

09:05 The Patient Voice – Living with Lobular Breast Cancer (B.C.)
Brenda Cunnington

09:20 Systemic therapy
Nancy Nixon

09:50 Surgical therapy
Andrea Covelli

10:05 Emerging Research
David Lim

10:20 Break with Exhibitors

Session VIX: Survivorship in Breast Cancer – Patient Advocate

Moderator(s): ***Melinda Wu***

10:50 Sexual Health and Breast Cancer
Michelle Jacobson

11:00 Breast Cancer During Pregnancy
Christine Elser

11:10 Exercise and Breast Cancer
Amy Kirkham

11:20 Optimal Imaging in Breast Cancer
Jean Seely

11:30 Moderated Panel Discussion with Audience

Session X: Best Poster Abstract Submission 2025:

Moderator(s): ***Amanda Roberts***

11:45 Noteworthy Abstracts 2025 – Scientific Committee's Third-Place Selection:
Upfront Tailored Axillary Surgery (TAS) for Clinically Node-Positive HR+/HER2- Breast Cancer: A Population-Based Cohort
Alison Laws

11:55 Outstanding Abstracts 2025 – Scientific Committee's Second-Place Selection:
Cost-Effectiveness of Breast Cancer Screening Using Digital Mammography in Canada
Moira Rushton

12:05 Top Abstracts 2025 – Scientific Committee's First-Place Selection:
10-Year Local Recurrence Rates Following Selective Omission of Re-excision for Patients with Ductal Carcinoma in Situ and Margins <2mm
Alison Laws

12:15 "People's Choice Award – Best Poster, Voted by Audience
Sexual HEalth and therapy adherence of estrogen receptor positive breast CANcer survivors on maintenance endocrine therapy – The SHE-CAN project in Toronto, Canada
Alliya Remtullatharani

12:30 Lunch and Networking

12:30 Non-Accredited Symposium

Session XI: Central Nervous System Metastases

Moderator(s): Moira Rushton

13:30 Radiation therapy
Hanbo Chen

13:45 Screening (Why or Why Not?)
Kasia Jerzak

14:00 Systemic therapy
Nathalie Levasseur

14:15 Break with Exhibitors

Session XII: Novel Advances in Breast Cancer

Moderator(s): ***Christine Simmons***

14:30 Novel Antibody Drug Conjugates
Mita Manna

14:45 Novel Imaging Techniques
Vivianne Freitas

15:00 Novel Immunotherapy/Cell Therapy
Mitchell Elliott

15:15 Moderated Panel Discussion with Audience

Session XIII: Advocacy in Breast Cancer

Moderator(s): ***Fahima Osman***

15:30 Breast Cancer Canada/REAL Alliance
Mita Manna

15:40 Rethink Breast Cancer
MJ DeCoteau

15:50 Patient Groups: Your Partners on the Ground
Aya McMillan – Patient Advocate
Rebecca Armstrong – *Canadian Breast Cancer Network*
Leila Springer – Olive Branch of Hope
Jenn Gordon – Rethink Breast Cancer

16:30 Closing Remarks
Christine Brezden-Masley & Tulin Cil
